# Hodgkin lymphoma in the bone marrow aspirate of a young patient with human immunodeficiency virus

**DOI:** 10.1002/jha2.670

**Published:** 2023-03-13

**Authors:** Evashin Pillay, Poobalan Naidoo

**Affiliations:** ^1^ University of KwaZulu‐Natal & National Health Laboratory Service, Inkosi Albert Luthuli Central & King Edward VIII Hospitals Durban South Africa; ^2^ University of KwaZulu‐Natal & KwaZulu Natal Provincial Department of Health Inkosi Albert Luthuli Central & King Edward VIII Hospitals Durban South Africa

**Keywords:** bone marrow aspirate morphology, HIV, Hodgkin lymphoma

1

A 31‐year‐old South African lady, HIV‐positive since 2019 (medication: fixed dose combination antiretroviral therapy – tenofovir, lamivudine and dolutegravir; CD4 count: 328 cells/µL; HIV viral load: undetectable) and who completed a full course of empiric therapy for tuberculosis (TB) 2 months prior, presented to the emergency room complaining of fatigue, loss of appetite and malaise; no bleeding.

During admission, her general examination was remarkable for muscle wasting, tachycardia, pallor, pyrexia (>38°C) and generalised significant lymphadenopathy (LAD), particularly evident as a left‐sided axillary mass. Systemic examination revealed a distended abdomen compatible with ascites, and reduced bi‐basal breath sounds; no hepatosplenomegaly. The overall clinical impression was that of a young female with stage 4 HIV and features of lymphoma, as supported by constitutional symptoms, LAD and serositis.

Blood work comprising an FBC demonstrated an isolated severe anaemia (leucocytes 5.20 × 10^9^/L – lymphocytes 7%, haemoglobin 5.1 g/dL and platelets 314 × 10^9^/L); corrected reticulocyte count 0.45%.

A staging CT (computed tomography) scan demonstrated generalised significant LAD (>1 cm; neck, supraclavicular, axillary (largest; >4 cm in short axis), mediastinal, hilar and para‐aortic), bilateral pleural effusions with bi‐basal atelectasis, gross ascites and hepatomegaly (±14 cm in span); no splenomegaly but multiple non‐enhancing round hypodensities were noted in the spleen.

Tissue biopsies were then planned, and the excision node histology confirmed a diagnosis of nodular sclerosis classic Hodgkin lymphoma (cHL), syncytial variant. A subsequent staging bone marrow (BM) biopsy was performed and remarkably, the BM aspiration smears revealed ∼5% large‐ and giant‐sized, mono‐ and multi‐nuclear tumour cells that morphologically resembled Hodgkin and Reed–Sternberg (HRS) cells (Figure 1). Unfortunately, several attempts to process the BM aspirate sample for flow cytometry proved unsuccessful due to repeated clogging of the sample injection tube. Nonetheless, the corresponding BM trephine biopsy sections showed focal infiltration by neoplastic cells, in addition to many pleomorphic counterparts (Figure 2). Immunohistochemical and special stains (Figure 2) revealed the HRS cells to express the following phenotype: CD45−, CD15+, CD30+, PAX5+, MUM1+, EBER+ (Epstein–Barr virus‐encoded RNA), CD3−, CD20−, CD138−, ALK− and AE1/AE3−. Moreover, identifiable in close proximity to the HRS cells were a pathognomonic mixed population of inflammatory cells (Figures 1 and 2). Trilinear haematopoiesis was relatively adequately preserved.

**FIGURE 1:**
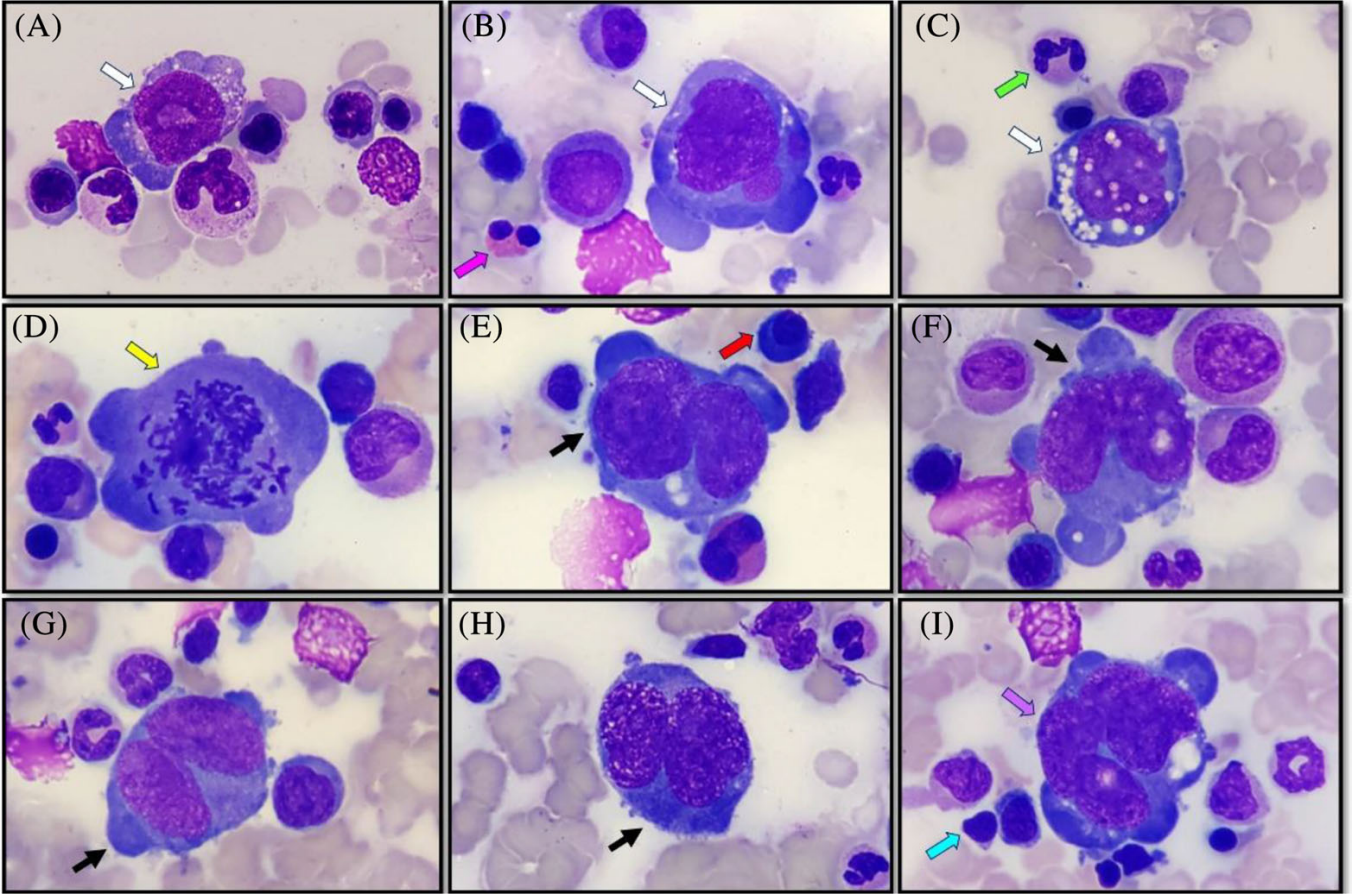
Bone marrow aspirate smear (panels A–I; May–Grünwald–Giemsa; 100× objective). Morphologically heterogeneous tumour cells including Hodgkin cells (panels A–C; white arrows), forms undergoing cell division (panel D; yellow arrow), Reed–Sternberg cells (panels E–H; black arrows) and multi‐nuclear counterparts (panel I; purple arrow) comprised of abundant, deeply basophilic, agranular cytoplasm with vacuolation and clasmatosis (pseudopod formation), and containing nuclei with irregular borders, reticulated chromatin, conspicuous nucleoli (panel A) and vacuolation (panels C, F and I). Inflammatory cells comprising eosinophils (panel B; pink arrow), granulocytes (panel C; green arrow), plasma cells (panel E; red arrow) and lymphocytes (panel I; cyan arrow) in close proximity to tumour cells.

**FIGURE 2:**
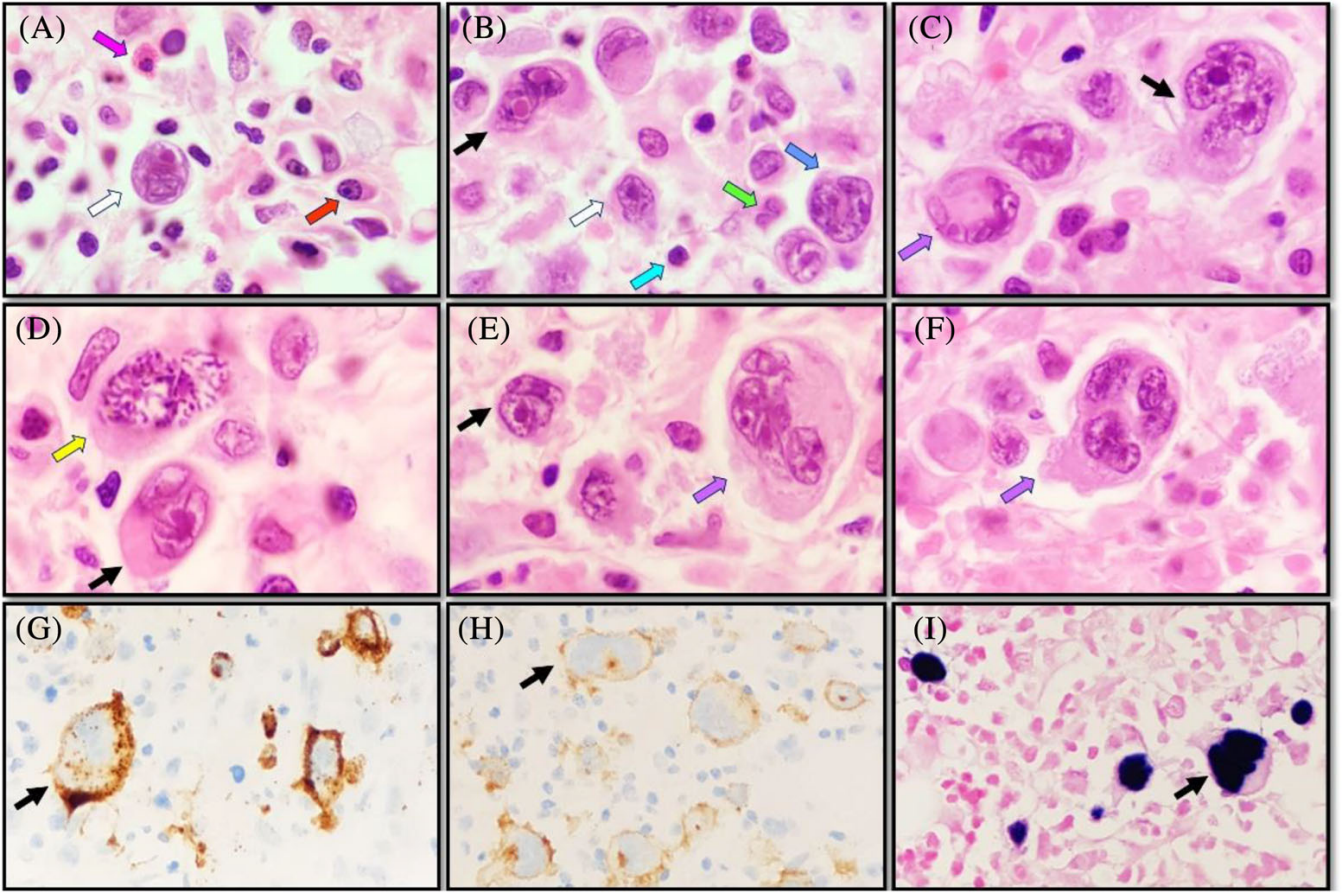
Bone marrow trephine sections (panels A–I; 100× objective). Hodgkin cells (panels A and B; haematoxylin and eosin [H&E]; white arrows), Reed–Sternberg cells (panels B–E; H&E; black arrows) and multi‐nuclear tumour cell variants (panels C, E and F; H&E; purple arrows). A lacunar cell (panel A; white arrow), hallmark‐like cell (panel B; blue arrow) and mitotic Reed–Sternberg cell with ‘cherry‐red’ nucleoli (panel D; yellow arrow). Immunostaining of tumour cells for CD15 (panel G; black arrow), CD30 (panel H; black arrow) and Epstein–Barr virus‐encoded RNA (EBER) (panel I; in situ hybridisation [ISH]; black arrow). Inflammatory cells comprising eosinophils (panel A; pink arrow), plasma cells (panel A; red arrow), granulocytes (panel B; green arrow) and lymphocytes (panel B; cyan arrow) in close proximity to tumour cells.

Finally, an international prognostic score of 4 was concluded (serum albumin 1.3 g/dL, haemoglobin 5.1 g/dL, stage IV disease and lymphocytes 7%). Notably, microbiological investigations for SARS‐CoV‐2 and TB were negative. Unfortunately, the patient succumbed to a hospital‐acquired pneumonia despite exhaustive supportive therapy and empiric antibiotics, and prior to initiating chemotherapy.

It is exceedingly rare to observe well preserved HRS cells in a BM aspirate film. The accompanying fibrotic milieu of the infiltrated BM probably causes tumour cell fragmentation and/or impairs their effective aspiration [1, 2, 3, 4]. Another consideration is that of scattered focal bony lesions which may not be biopsied during the process of attempting aspiration [5, 6].

This case highlights an exceptional clinicopathologic entity by demonstrating HRS cells in the BM aspirate film of a young HIV‐positive patient presenting with the syncytial variant of nodular sclerosis cHL. It has enabled careful examination of numerous HRS cells thereby contributing to appreciation of their morphological heterogeneity. Haemato‐pathologists should be cognizant of such cases to prevent misdiagnosis as metastatic non‐haematological neoplasms (particularly carcinoma) or other haematological neoplasms (particularly non‐Hodgkin lymphoma) [7, 8]. Accordingly, timeous diagnosis may prove to be lifesaving, especially in resource‐constrained settings.

## CONFLICT OF INTEREST STATEMENT

The authors declare no conflict of interest.

## ETHICS STATEMENT

Ethical approval was obtained from the Biomedical Research Ethics Committee (BREC) affiliated with the University.

## Data Availability

The data that support the findings of this study are available from the corresponding author upon reasonable request.
